# One-year follow-up of B vitamin and Iron status in patients with phenylketonuria provided tetrahydrobiopterin (BH4)

**DOI:** 10.1186/s13023-018-0923-2

**Published:** 2018-10-30

**Authors:** Kristen D Brantley, Teresa D Douglas, Rani H Singh

**Affiliations:** 1 Emory University Rollins School of Public Health, Atlanta, GA USA; 2 Department of Human Genetics, Metabolic Nutrition Program, Emory University School of Medicine, Atlanta, GA USA

**Keywords:** Phenylketonuria, Tetrahydrobiopterin (BH4), Sapropterin, B-vitamin deficiency, Iron deficiency

## Abstract

**Background:**

People with Phenylketonuria (PKU) who respond to tetrahydrobiopterin (BH4) often decrease dependence on medical food (MF) following increased phenylalanine (phe) tolerance. Responders to BH4 may experience a reduction in certain nutrients if not compensated through intact foods or supplements. This study investigated B6, B12, folate, and iron status based on blood levels and dietary intake in patients with PKU responsive to BH4 over 1 year.

**Methods:**

Fifty-eight patients with PKU, ages 4–50 years were recruited and initiated on BH4 therapy. Patients were monitored for BH4 response, and nutritional status was recorded at regular intervals over 12 months. The analysis included 33 patients with known BH4 response status and complete nutritional data. Nutrient intake was determined by National Data System for Research (NDSR) analysis of self reported 3 day diet records and compared to Dietary Reference Intakes (DRIs). Blood biomarkers were analyzed by Quest Diagnostics and compared to laboratory reference ranges. Patient laboratory values were compared to controls from the National Health and Examination Survey (NHANES). Differences in nutrient intakes across time points were examined, stratified by age, using nonparametric methods. Statistical analyses were completed with SAS 9.4, with significance set at α = 0.05.

**Results:**

Medical food intake declined among pediatric (*p* < 0.01) and adult (*p* = 0.06) BH4 responders over 1 year. Among those < 18 years of age, mean percent of calories obtained from MF declined from 21.3 to 4.7%. In adults, percent calories from MF dropped from 19.5 to 4.0%. Though maintaining laboratory and dietary values within reference ranges, responders < 18 years experienced a significant decline in serum B12 (*p* = 0.01), dietary folate (*p* = 0.006), and dietary iron (*p* = 0.004) over the study.

**Conclusion:**

Although mean dietary and laboratory values for B12, B6, folate, and iron in BH4 responders and non-responders were adequate at baseline and 12-month follow-up, responders experienced a significant decline in serum B12 over 1 year, which may be explained by decreased intake of fortified MF. Both response groups had lower serum B12 than NHANES controls at baseline and 12 months. Results indicate a need to monitor B12 concentrations and consider micronutrient supplementation, with special attention to pediatric patients with PKU.

**Electronic supplementary material:**

The online version of this article (10.1186/s13023-018-0923-2) contains supplementary material, which is available to authorized users.

## Background

Phenylketonuria (PKU) is an autosomal recessive inborn error of metabolism identified in infancy through newborn screening. This disorder can lead to severe neurological or psychomotor complications if left untreated [1]. PKU is effectively controlled by maintaining blood phenylalanine (phe) levels within therapeutic range (120 to 360 μmol/L), which can be accomplished with dietary restriction of phe along with intake of a phe-free amino acid mixture (AAM), or “medical food” (MF), as the key protein source [2–4]. Patients who are responsive to tetrahydrobiopterin (BH4), an enzyme cofactor necessary for the catalysis of phe to tyrosine (tyr) by phenylalanine hydroxylase (PAH), may benefit from BH4-driven therapy such as sapropterin dihydrochloride (Kuvan®) [5]. Sapropterin, approved by the FDA in 2007 and deemed adequately safe [6, 7], has been shown to lower blood phe concentrations and increase dietary phe tolerance in the BH4 responsive subset of patients with PKU, thus allowing for some diet liberalization [3, 6, 8].

While sapropterin is an effective adjunct therapy for many PKU patients, the consequent diet liberalization often results in decreased requirement for MF consumption [3, 8, 9]. The limited increase in nutrient intake from intact foods following diet liberalization, especially nutrients primarily derived from animal protein sources including B12 and iron [10], may not be sufficient to account for the corresponding loss of nutrients from decreased MF [11].

Recent studies have revealed the potential for macro- and micronutrient deficiencies in BH4-responsive patients [8, 11, 12]. Others have reported potential B6 and B12 deficiencies based on diet or lab analysis among PKU patients following liberalization of diet, irrespective of BH4 status [13–16]. However, the clinical significance of decreases in micronutrient status has not been determined, and nutrients of concern vary from study to study [8, 11, 17]. It is therefore essential to determine the extent that PKU responders to BH4 are at risk of nutritional deficiencies and to identify their potential need for supplementation.

Given prior evidence of B-vitamin deficiencies among patients with PKU on liberalized diets, and the biologic mechanisms which link B-vitamins and iron to erythroblast production and anemia risk [18], we evaluated B-vitamin and iron nutritional status among patients with PKU before and 1 year on BH4. We hypothesized that BH4 responders would experience a change in serum B12, folate, and total iron along with plasma B6 concentrations resulting from reduced MF intake.

Prior studies evaluating micronutrient status in patients with PKU responsive to BH4 have been limited by reliance on diet records alone [12, 16], have had small sample sizes with limited age ranges [11], and have taken place in European populations [11, 12] where dietary patterns may differ compared to U.S. norms. To address these limitations our study evaluated serum B12, folate, total iron, and plasma B6 concentrations in tandem with dietary intakes of these key nutrients in an age and gender diverse sample of patients with PKU in the U.S.

## Methods

### Study design

The study protocol and schematic has been described elsewhere in detail [9, 19]. Briefly, 58 patients with PKU, 4 years of age and older, were recruited between October 2008 and September 2009 through Emory University Department of Human Genetics Healthcare Clinic (Atlanta, GA). Inclusion criteria included patients with PKU naïve to BH4 treatment, not involved in other clinical trials, and age 4 years and above. Exclusion criteria included pregnancy or planning to become pregnant, breastfeeding, not diagnosed with PKU, and inability to provide informed consent. All participants were given 20 mg/kg/day of sapropterin dihydrochloride (Kuvan®, Biomarin Pharmaceutical Inc) for 1 month. Patients were assigned as preliminary responders if they experienced a decrease in plasma phe during the initial treatment month of at least 15%, and if they continued sapropterin treatment throughout the study period. Non-responders were removed from BH4 after the one-month trial and returned to their standard treatment plan. A dietary phe challenge to adjust for BH4-dependent changes in phe tolerance [20] set apart definitive responders (improved blood phe control combined with increased dietary phe tolerance) from provisional BH4 responders who experienced an initial lowering of plasma phe but no substantial long-term change in phe tolerance, MF requirement, or blood phe control [9, 19]. Patient PAH genotypes are presented in a prior publication with the assigned value (AV) sum provided [21].

Each patient was followed for up to 1 year. Data were collected at baseline and at three consecutive study visits spaced evenly throughout the year, with final data collection completed at 1 year. Three day diet records were completed at each study visit and analyzed by a registered dietitian. Average energy, phe (mg), protein (g), amino acids (g), fatty acids, and macro- and micronutrient intake were determined using the Nutrition Data System for Research (NDSR, University of Minnesota) diet analysis software, and reported as units per day. A blood draw was also performed at each visit for laboratory analysis of plasma amino acids (Emory Genetics Lab, Amino Acid Analyzer) and micronutrient biomarkers (Quest Diagnostics). Table 1 lists specific blood biomarkers for each micronutrient investigated.

**Table 1:** Micronutrient Laboratory Biomarkers and Reference Ranges

Micronutrient	Biomarker, analysis method	Units	Plasma or serum	Reference ranges^a^	
B6	Pyridoxal phosphate (PLP), LC/MS/MS	ng/mL	Plasma	2–17 Years	3.0–35.0 ng/mL
				Adult	2.1–21.7 ng/mL
B12	Cobalamin, Immunoassay	pg/mL	Serum	5–9 Years	200–1205 pg/mL
				10–17 years	260–935 pg/mL
				> 17 years	200–1100 pg/mL
Folate	Folate, Immunoassay	ng/mL	Serum	5–9 years	> 7.1 ng/mL
				10–17 years	> 8.0 ng/mL
				≥18 years	> 5.4 ng/mL
Iron (total)	Iron, Spectrophotometry	mcg/dL	Serum	Both sexes	
				4–19 years	27–164 mcg/dL
				Male	
				20–29 years	50–195 mcg/dL
				≥ 30 years	50–180 mcg/dL
				Female	
				20–49 years	40–190 mcg/dL
				≥ 50 years	45–160 mcg/dL

^a^Quest Diagnostics

### NHANES data collection

To compare laboratory nutrient values in our sample with a standard population, two age and gender matched controls were selected for each patient from the most recent NHANES collection with available B12, B6, folate, and iron blood measurements (NHANES 2005–2006). NHANES serum and plasma based laboratory measurements were performed following Center for Disease Control and Prevention (CDC) guidelines [22].

### Statistical analysis

All statistical procedures were completed using SAS software v.9.4.1 [SAS Institute Inc., Cary, NC, USA]. Because dietary recommended intakes (DRI) differ based on age, analyses were stratified by age group (< 18 years and ≥ 18 years). Baseline and final visit time points were used in assessment. Where final visit dietary data was not recorded, and where participants had stabilized diets by the previous visit (month 8), data from month 8 was used to represent the final visit (*n* = 7). Participants with missing diet or laboratory information were excluded from analysis. Provisional responders were also removed from the analysis to allow clear comparison of diet-liberalized responders on BH4 to non-responders.

Distributions for all dependent variables were tested for normality in each age strata using graphical interpretation and the Kolmogorov–Smirnov test. Several baseline or endpoint values deviated substantially from normality; given our small sample size, non-parametric two-tailed tests were chosen for analyses to maintain consistency. For all procedures, significance was tested at an alpha level of 0.05.

Correlations between laboratory values and reported dietary intake were determined using Spearman rho correlations. Significant changes in laboratory and/or dietary nutrient values were assessed using the Wilcoxon Signed Rank Test, with average values used for ties. Differences between BH4 response groups at baseline and final visit were evaluated via the Wilcoxon-Mann-Whitney test (NPAR1WAY procedure in SAS) for each age group. The Kruskal-Wallis test was conducted to compare baseline and end point values in each response group with NHANES controls.

The percentage of nutrients obtained from MF and intact food was calculated from diet record data at baseline and final visit. MF intake included primary and secondary formula, if prescribed. Differences in the percentage of micronutrients obtained from intact foods versus MF were tested using the Wilcoxon-Mann-Whitney and Signed Rank tests.

## Results

### Participant characteristics

**Fig. 1:**
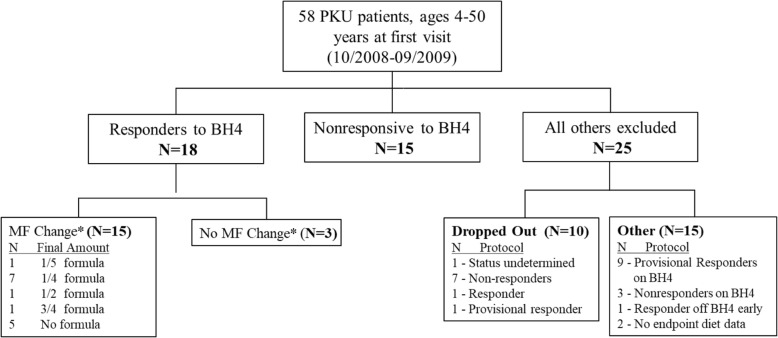
Participant Flowchart for PKU Kuvan Study, from 2008 to 2009 Baseline Enrollment. *MF change indicates prescription at end of study as a fraction of the MF prescription at baseline. The five reported as “No formula” had all prescribed MF removed from their PKU diet by study end

In total, 48 of the 58 recruited patients completed the one-year study. The final sample for analysis included 33 subjects, 15 non-responsive to sapropterin therapy and 18 confirmed to be responsive, henceforth referred to simply as “responders”. Exclusions are described in Fig. 1.

In the final analysis cohort, the mean age was 18.8 years, with 19 (57.5%) < 18 years of age and 14 (42.4%) ≥18 years of age. The majority of participants were female (51.5%) (Table 2). At baseline gender, anthropometrics and MF adherence, defined as amount of MF consumed below prescribed, were not significantly different between response groups. Responders were younger on average than non-responders (16.6 years, SD = 10.3 v. 21.5 years, SD = 14.2). Approximately 60% of participants reported taking the full amount of prescribed MF in both groups.

**Table 2:** Participant Characteristics at Baseline, Responders v. Non-responders (*N* = 33)

Variable (baseline)	< 18 years		≥18 years	
	Responder (*n* = 11) n (%) or mean (sd)	Non-responder (*n* = 8) n (%) or mean (sd)	Responder (*n* = 7) n (%) or mean (sd)	Non-responder (*n* = 7) n (%) or mean (sd)
Age (years)	9.7 (3.4)	10.4 (4.4)	27.4 (7.6)	34.2 (9.7)
Sex				
Male, n (%)	8 (73)	3 (37)	2 (29)	3 (43)
Female, n (%)	3 (27)	5 (63)	5 (71)	4 (57)
Average weight (kg)	34.8 (19.2)	46.4 (27. 8)	72.4 (17.2)	76.4 (20.8)
Average height (cm)	138.7 (21.2)	138.4 (23.8)	168.2 (10)	165.3 (7.8)
MF adherence n (%)				
Take full amount	7 (64)	7 (88)	4 (57)	2 (29)
Do not take full amount	3 (27)	0	2 (29)	3 (43)
N/A	0	0	1 (14)	0
Missing	1 (9)	1 (12)	0	2 (29)
Total Calories (kcal)	1556 (321)	1571 (344)	2354 (830)	1812 (275)
Taking two MF				
Yes	2 (18.2)	3 (37.5)	1 (14.3)	2 (28.6)
No	9 (81.8)	5 (62.5)	6 (85.7)	5 (71.4)
Percent of total protein intake from MF^b^	54.1 (35.2)^a^	76.5 (13.8)^a^	49.3 (29.5)	59.2 (29.1)

*N/A* not applicable, *sd* standard deviation

^a^Mean intake of MF in g significantly different between responders/non-responders at baseline for < 18 years (*p* = 0.02)

^b^Includes protein intake from both primary and secondary MF

### Plasma phe control and dietary changes

After 1 year of follow-up, median plasma phe decreased among BH4 responders while phe obtained from intact foods increased slightly (Table 3), indicating improved phe tolerance. These long term changes did not occur among non-responders. The percent of calories obtained from MF and the amount of prescribed MF was greatly reduced among responders by the end of study (Table 3, Fig. 1).

**Table 3:** Plasma phe and reported phe intake from intact foods at start and end of study, BH4 responders (*N* = 18)

Measure	Baseline	End of Study	*p*-value for change^a^
	Median (IQR)	Median (IQR)	
Plasma phe (umL)	461.5 (366, 539)	355 (231, 427)	0.048
Intact food phe (mg/day)	791 (529, 2207)	1198 (993, 1457)	0.535
Percent calories from MF	20.0 (9.08, 30.18)	0 (0, 10.2)	0.002

^a^Testing significant change over one-year, using signed rank test, α =0.05

The result of greater phe tolerance among responders in this study was liberalization from a diet where intact food is restricted with measured amounts of fruits, vegetables, and specialty low-protein foods to a diet with more liberal intake of grains, legumes, nuts, fruits, vegetables, and limited amounts of animal protein, as seen in other studies [11, 20, 23].

### BH4 responders experienced declines in micronutrient labs over 1 year

**Fig. 2:**
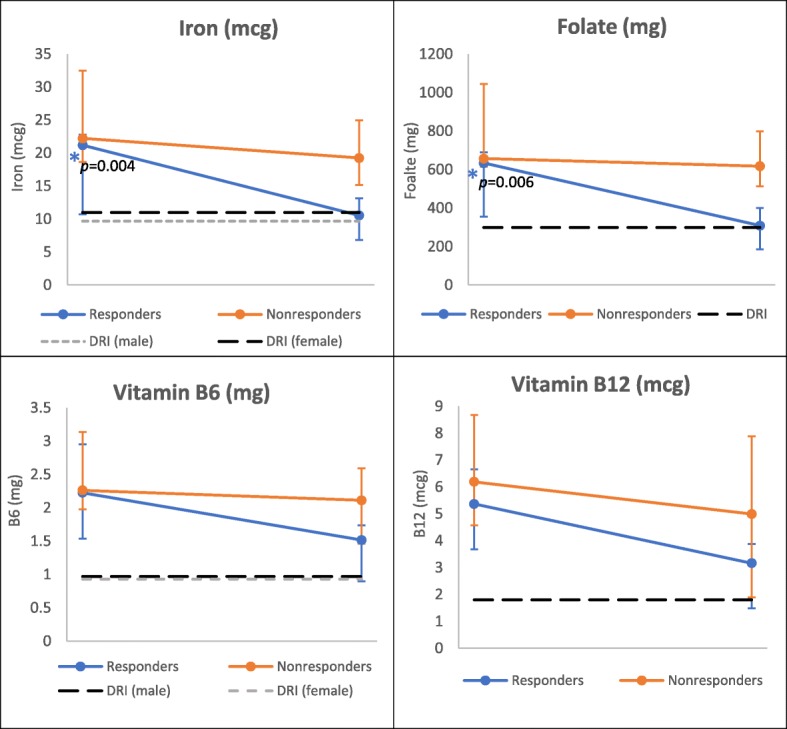
Change in Dietary Micronutrients over One Year by Response Group, < 18 years. *Indicates statistically significant change over one-year, using signed rank test, α =0.05. *P* values for change over time (responders): iron *p* = 0.004; folate *p* = 0.006

**Fig. 3:**
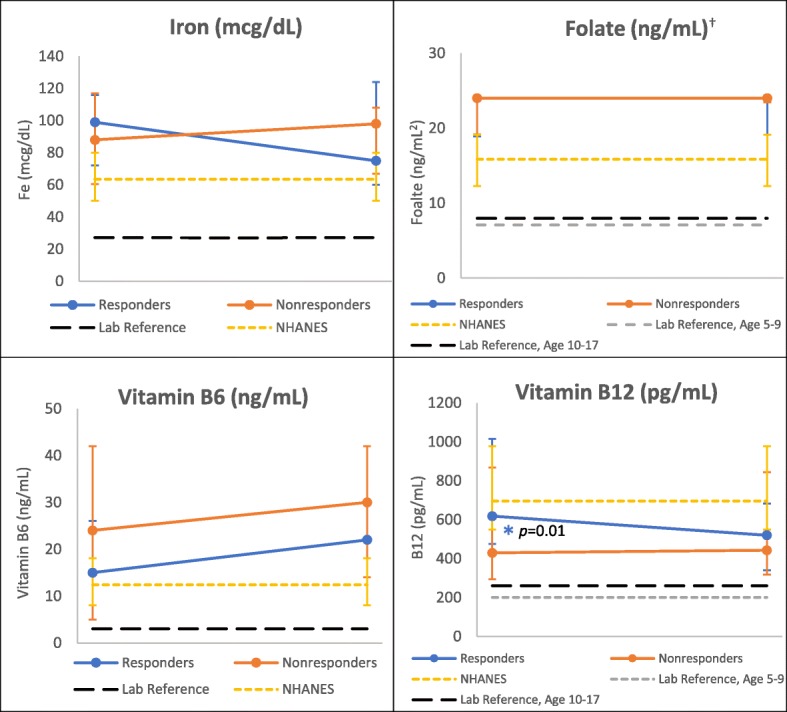
Change in Laboratory Micronutrients over One Year by Response Group, < 18 years. ^δ^ NHANES data represents a single time point of collection and laboratory reference given as lower limit. * Indicates statistically significant change over one-year, using signed rank test, α =0.05. Vitamin B12 *p* value for change from start to end (responders) = 0.01. † Folate above 24 ng/mL was recorded as 24 ng/mL

**Fig. 4:**
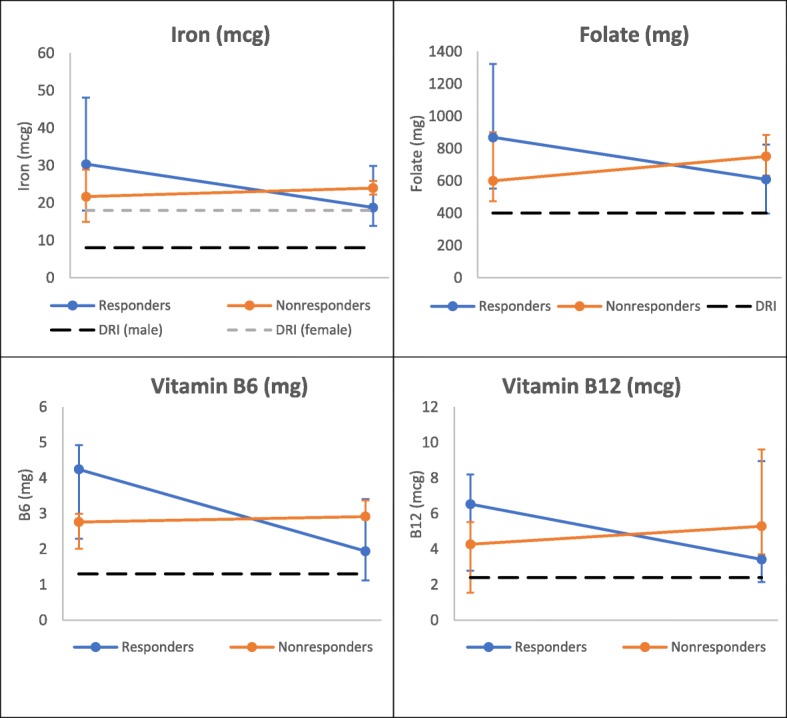
Change in Dietary Micronutrients over One Year by Response Group, ≥18 years

**Fig. 5:**
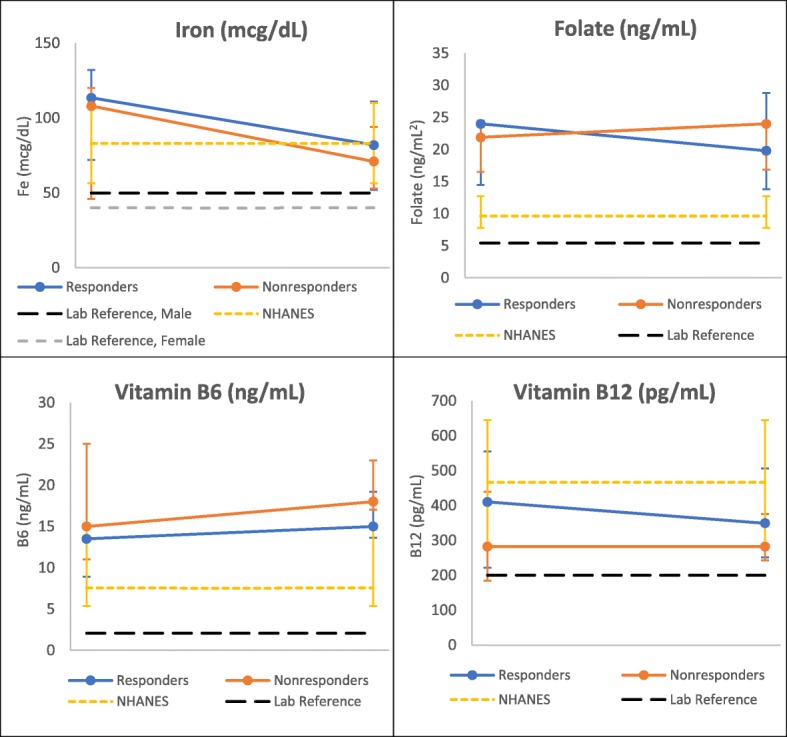
Change in Laboratory Micronutrients over One Year by Response Group, ≥18 years. ^δ^ NHANES data represents a single time point of collection and laboratory reference given as lower limit

All BH4 responders had lower dietary and serum-assessed iron, folate, and B12 after 1 year, compared to baseline (Figs. 2, 3, 4 and 5, Additional file 1: Table S1 and S2), though plasma B6 increased over the same period in both age groups. When stratified by age, the 1 year decline observed in dietary folate and iron remained statistically significant among responders < 18 years of age (Fig. 2). Furthermore, those within the lower quartile of intake fell below DRI ranges after 1 year. Serum B12 also dropped significantly among responders in the younger age group over the 1 year study period (Fig. 3). While pediatric responders had comparable serum micronutrient levels to NHANES controls at baseline, at the end of study B12 fell significantly below NHANES control values (*p* = 0.03) (Fig. 3). Overall serum iron also declined among young responders over the study period, driven by significant declines in serum transferrin, though total levels remained higher than NHANES controls.

Among BH4 non-responders, dietary micronutrients remained consistent from baseline to end of study (Additional file 1: Table S1). Serum-measured micronutrients increased on average among non-responders from baseline to end of study in both age groups (Additional file 1: Table S2).

### Change in micronutrients may be related to MF intake

Responders in both age groups experienced a decline in proportion of caloric and nutrient intake derived from MF over the one-year period (Table 4). This decline was statistically significant for all nutrients considered among those < 18 years of age (*p* = 0.02). Overall declines in micronutrients may be attributed to the lowered MF intake, especially given comparison with non-responders and older responders who did not see significant declines in percentage MF intake or laboratory and dietary measured micronutrients over time. AV sum [21] was also investigated as a potential source of micronutrient change, though it was not a significant factor in either laboratory or dietary micronutrient outcomes.

**Table 4:** Proportion of Nutrient Intake from MF at Baseline and End of Study

Nutrients	< 18 years				≥18 years			
	Responders		Non-responders		Responders		Non-responders	
	Mean % (sd) BL/End	*P* value^a^ (change)	Mean % (sd) BL/End	*P* value^a^ (change)	Mean % (sd) BL/End	*P* value^a^ (change)	Mean % (sd) BL/End	*P* value^a^ (change)
Calories from MF^b^	21.3 (16.4)/ 4.7 (6.0)	0.008	36.1 (10.2)/ 27.0 (20.1)	0.25	19.5 (14.1)/ 4.0 (9.0)	0.06	23.5 (18.3)/ 30.1 (29.6)	0.81
Vitamin B6	38.63 (31.0)/ 12.3 (16.6))	0.02	69.6 (11.6)/ 55.7 (34.8)	0.46	25.4 (26.9)/9.4 (21.2)	0.43	44.7 (33.6)/ 50.7 (37.7)	0.63
Vitamin B12	47.1 (38.2)/ 22.2 (30.7)	0.02	87.4 (11.1)/ 69.6 (43.7)	0.64	47.1 (41.4)/ 13.1 (31.1)	0.13	62.3 (45.1)/ 61.9 (40.5)	1.0
Folate	46.7 (36.3)/ 15.9 (21.1)	0.02	76.5 (13.2)/ 62.1 (39.3)	0.38	40.2 (35.8)/11.1 (24.4)	0.13	49.5 (36.6)/ 56.8 (40.9)	1.0
Iron	43.2 (34.4)/ 13.1 (16.0)	0.02	74.1 (12.3)/ 59.3 (37.3)	0.38	42.1 (34.9)/ 11.8 (26.1)	0.13	46.6 (35.2)/ 57.8 (39.9)	1.0

*BL* baseline, *MF* medical food, *sd* standard deviation, ^a^ *p* value for change over time obtained from signed rank test, α = 0.05

^b^ Combines primary and secondary MF in calculation of % intake from MF

### Most participants met DRI and laboratory reference values

Median micronutrient values remained above DRI and laboratory reference ranges among responders despite noted declines over the study period. In addition, the number of responders who did not meet DRI values increased from start to end of study for all micronutrients (Table 5). For instance, only one responder reported dietary B12 below their DRI level at the start of the study, whereas six responders reported insufficient dietary B12 at the end of the study. Most responders had micronutrient values that fell within laboratory reference ranges, with no shifts outside of acceptable ranges seen from start to end of study (Table 5).

**Table 5:** Number of Responders Meeting and not Meeting DRI and Laboratory Reference Ranges

Micronutrient^c^	Dietary Reference^a^				Laboratory Reference^a^			
	Responders		Non-responders		Responders		Non-responders	
	# met / # not met^b^		# met / # not met^b^		# met / # not met^b^		# met / # not met^b^	
	Baseline	Endpoint	Baseline	Endpoint	Baseline	Endpoint	Baseline	Endpoint
Vitamin B6	15/2	12/5	14/1	12/2	17/0	18/0	14/0	12/0
Vitamin B12	16/1	11/6	13/2	11/3	16/2	17/1	13/2	13/2
Folate	14/3	10/7	15/0	12/2	11/0^d^	18/0	14/0	15/0
Iron	13/4	10/7	14/1	13/1	17/0	17/1	13/2	14/1

*DRI* dietary reference intake

^a^Dietary reference met based on reported 3-day diet record analysis. Laboratory reference met based on measurements from Quest Diagnostics, assessed as above minimum level

^b^Missing values not reported

^c^No statistically significant difference between no. meeting DRI guidelines at start vs. endpoint, by Fisher’s exact test, *p* = 0.05

^d^7 missing values for laboratory folate at baseline due to laboratory error

### Micronutrient intake was lower among responders vs. non-responders at end of study

At baseline, there were no significant differences in dietary nutrient values between response groups within either age group. After 1 year, responders in both age groups had lower dietary levels compared to non-responders of all assessed micronutrients (Figs. 2 and 4). In the younger age group, there were significant differences between responder and non-responder dietary intakes at endpoint for iron (*p* = 0.02), folate (*p* = 0.004), and B6 (*p* = 0.04). Laboratory assessed micronutrients appeared similar between both groups at individual time points (Figs. 3 and 5), though iron levels among responders did drop below non-responders in those under 18 years (Fig. 3).

## Discussion

In this study of 33 patients with PKU followed over the course of 1 year, there was a marked decline in serum-measured B12, dietary folate and iron among BH4-responsive patients under age 18. This result may be explained by a decline in the proportion of B-vitamins and iron that responders obtained from MF from start to end of study, which supports our initial hypothesis.

Although we did not observe many deficiencies in serum-measured micronutrients, the decline in B12 over time seen in our study does correspond with evidence from previous studies that observed B12 deficiencies or borderline deficiencies among PKU patients under BH4 therapy [13], and on relaxed [16, 24], or unrestricted diets [14]. Our results match those seen in a study of German patients with PKU, which observed no deficiency in B12 among patients with PKU regardless of MF intake but did identify a positive correlation between B12 and other micronutrients and the amount of protein obtained from MF [12].

It is interesting that declines in micronutrient intake were more notable among the younger age group as opposed to the older age group, especially given typically higher non-adherence with MF among adults [25, 26]. The dampened impact in those over 18 years may be explained by larger variations in nutrient intake in this group, likely resultant from the wide age range (18–50 years). Thus, the overall decline in intake observed among adult patients may be clinically relevant despite lacking statistical significance.

Clinician monitoring appeared to be effective in helping most patients maintain sufficient micronutrient intake. Multivitamin/mineral supplementation was recommended to the majority confirmed as BH4 responders, as well as patients not adherent to their prescribed MF.

This study benefits from the relatively large sample size considering the rarity of PKU. We were able to evaluate dietary reported intake as well as laboratory plasma and serum biomarkers for an accurate measure of patients’ iron and B-vitamin status. Laboratory assessment is especially important given the oftentimes low adherence to prescribed MF seen among patients with PKU, especially adults [25, 26]. Additionally, patients may neglect instructions for recommended vitamin/mineral supplements or not capture important dietary details in their 3 day food records.

Despite our relatively large sample size, we were limited by small sample numbers within each age strata. Although all BH4 responders were instructed by our dietitians to begin a multivitamin/mineral supplement as MF was reduced and log this in their diet records, most patients neglected these instructions. Because the records did not reliably capture multivitamin and mineral intake, we were unable to assess their nutritional contribution. This may have contributed to lack of correlations between laboratory and diet record reported micronutrient values. Further studies should closely monitor patient multivitamin and mineral intake from all sources to determine the degree supplementation may be required. Even so, observed declines in micronutrients from both diet and lab analyses indicate the importance of monitoring nutritional status long term in BH4 responders. Additional studies assessing changes beyond 1 year may be needed to determine whether micronutrient levels will remain stable and above reference ranges over a longer period or continue declining. Retrospective chart reviews of study patients who have continued care with Emory Genetics may provide the data for longer term analysis.

## Conclusion

Overall, patients with PKU receiving BH4 therapy may experience a decrease in micronutrient intake after decreasing intake of MF, their primary source of protein and micronutrient nutrition. Clinic-based monitoring appears to be effective in deterring nutrient deficiencies. Based on notable changes in B12 over time among those in our study on BH4 therapy, and significant declines in micronutrient intakes among the pediatric group, we encourage continuous long term monitoring of patients on liberalized diet to ensure adequate micronutrient status, with recommendation of a multivitamin/mineral supplement if clinically indicated. Further exploration of micronutrient status will help determine how to best avoid any deficiencies during dietary transitions.

## Additional file

Additional file 1: **Table S1.** Differences in Dietary Micronutrient Values, at Start and Endpoint, by Response Status. **Table S2.** Differences in Laboratory Micronutrient Values, at Start and Endpoint, by Response Status. (DOCX 24 kb)
