# Supplementary material for: One-year follow-up of B vitamin and Iron status in patients with phenylketonuria provided tetrahydrobiopterin (BH4)
Source: Orphanet J Rare Dis. 2018 Oct 30;13:192. doi: 10.1186/s13023-018-0923-2 (PMC6206913; doi:10.1186/s13023-018-0923-2)
Supplement: Supplementary file 1 — Table S1. Differences in Dietary Micronutrient Values, at Start and Endpoint, by Response Status. Table S2. Differences in Laboratory Micronutrient Values, at Start and Endpoint, by Response Status. (DOCX 24 kb) [file 13023_2018_923_MOESM1_ESM.docx]

**Additional file 1**

| **Table S1. Differences in Dietary Micronutrient Values, at Start and Endpoint, by Response Status** | | | | | | | |
| --- | --- | --- | --- | --- | --- | --- | --- |
|  | **<18 Years** |  |  |  | **≥18 years** |  |  |
| **Micronutrient** | Responders  Median (Q1, Q3) | Nonresponders Median (Q1, Q3) | *P* value† |  | Responders  Median (Q1, Q3) | Non-responders  Median (Q1, Q3) | *P* value† |
| *Baseline* |  |  |  |  |  |  |  |
| Iron (mg) | 21.2 (10.7, 22.8) | 22.2 (18.7, 32.5) | 0.20 |  | 30.4 (18.0, 48.1) | 21.7 (15.0, 28.9) | 0.13 |
| B6 (mg) | 2.2 (1.5, 3.0) | 2.3 (2.0, 3.1) | 0.50 |  | 4.2 (2.3, 4.9) | 2.8 (2.0, 3.0) | 0.13 |
| B12 (mcg) | 5.4 (3.7, 6.7) | 6.2 (4.6, 8.7) | 0.35 |  | 6.5 (2.8, 8.2) | 4.3 (1.5, 5.5) | 0.25 |
| Folate (mcg) | 634 (356, 689) | 657 (614, 1044) | 0.20 |  | 870 (554, 1324) | 601 (475, 902) | 0.25 |
| *Endpoint* |  |  |  |  |  |  |  |
| Iron (mg) | 10.5 (6.8, 13.1) | 19.2 (15.2, 24.9) | 0.01 |  | 18.8 (13.9, 29.9) | 24.0 (22.2, 25.9) | 0.81 |
| B6 (mg) | 1.5 (0.9, 1.7) | 2.1 (1.5, 2.6) | 0.04 |  | 1.9 (1.1, 3.4) | 2.9 (2.9, 3.4) | 0.47 |
| B12 (mcg) | 3.2 (1.5, 3.9) | 5.0 (1.9, 7.9) | 0.27 |  | 3.4 (2.1, 9.0) | 5.3 (3.7, 9.6) | 0.58 |
| Folate (mcg) | 309 (186, 400) | 617.5 (512, 798) | 0.004 |  | 609 (397, 825) | 752 (633, 885) | 0.69 |
| Q1, Q3=interquartile range  †Two-sided p value from Wilcoxon-Mann-Whitney test, α=0.05. | | | | | | | |

| **Table S2. Differences in Laboratory Micronutrient Values, at Start and Endpoint, by Response Status** | | | | | | | |
| --- | --- | --- | --- | --- | --- | --- | --- |
|  | **<18 Years** |  |  |  | **≥18 years** |  |  |
| **Micronutrient** | Responders  Median (Q1, Q3) | Nonresponders Median (Q1, Q3) | *P* value† |  | Responders  Median (Q1, Q3) | Non-responders  Median (Q1, Q3) | *P* value† |
| *Baseline* |  |  |  |  |  |  |  |
| Iron (mcg/dL) | 99 (72, 116) | 88 (60.5, 117) | 0.65 |  | 114 (72, 132) | 108 (46, 120) | 0.52 |
| B6 (ng/mL) | 15 (8.0, 26) | 24 (5.0, 42) | 0.52 |  | 13.5 (4.0, 14) | 15 (9.0, 24) | 0.47 |
| B12 (pg/mL) | 618 (475, 1015) | 430 (294, 869) | 0.30 |  | 410 (222, 555) | 282 (265, 388) | 0.52 |
| Folate (ng/ml^2^) | 24 (18.9, 24) | 24 (19.2, 24) | 0.88 |  | 24 (19.4, 24) | 21.9 (16.5, 24) | 0.37 |
| *Endpoint* |  |  |  |  |  |  |  |
| Iron (mcg/dL) | 75 (60, 124) | 98 (67, 108) | 0.59 |  | 82 (52, 111) | 71 (53, 94) | 0.95 |
| B6 (ng/mL) | 22 (14, 30) | 30 (14, 42) | 0.50 |  | 15 (11, 25) | 18 (17, 23) | 0.74 |
| B12 (pg/mL) | 520 (341, 683) | 443 (317, 844) | 0.97 |  | 349 (251, 506) | 282 (243, 375) | 0.31 |
| Folate (ng/ml^2^) | 24 (19.1, 24) | 24 (23.5, 24) | 0.42 |  | 19.8 (18.4, 24) | 24 (16.9, 24) | 0.68 |
| Q1, Q3=interquartile range  †Two-sided p-value from Wilcoxon-Mann-Whitney test, α=0.05. | | | | | | | |
